# Carbon Monoxide Targeting Mitochondria

**DOI:** 10.1155/2012/749845

**Published:** 2012-03-20

**Authors:** Cláudia S. F. Queiroga, Ana S. Almeida, Helena L. A. Vieira

**Affiliations:** ^1^Instituto de Biologia Experimental e Tecnológica (IBET), Apartado 12, 2781-901 Oeiras, Portugal; ^2^Instituto de Tecnologia Química e Biológica (ITQB), Universidade Nova de Lisboa, Apartado 127, 2781-901 Oeiras, Portugal; ^3^CEDOC, Faculdade de Ciência Médicas, Universidade Nova de Lisboa, 1169-056 Lisboa, Portugal

## Abstract

Mitochondria present two key roles on cellular functioning: (i) cell metabolism, being the main cellular source of energy and (ii) modulation of cell death, by mitochondrial membrane permeabilization. Carbon monoxide (CO) is an endogenously produced gaseoustransmitter, which presents several biological functions and is involved in maintaining cell homeostasis and cytoprotection. Herein, mitochondrion is approached as the main cellular target of carbon monoxide (CO). In this paper, two main perspectives concerning CO modulation of mitochondrial functioning are evaluated. First, the role of CO on cellular metabolism, in particular oxidative phosphorylation, is discussed, namely, on: cytochrome *c* oxidase activity, mitochondrial respiration, oxygen consumption, mitochondrial biogenesis, and general cellular energetic status. Second, the mitochondrial pathways involved in cell death inhibition by CO are assessed, in particular the control of mitochondrial membrane permeabilization.

## 1. Introduction

Carbon monoxide (CO) is a colorless and odorless small molecule, widely known as a lethal gas and as a toxic air pollutant. CO toxicity was disclosed in 1912 by Douglas [1]; its high affinity for haemglobin, forming carboxyhaemglobin, compromises oxygen delivery in tissues and subsequently causes lethality. Several decades later, CO was found as an endogenous generated gas in humans [2, 3]. However, only in the late sixties, haem oxygenase (HO) was characterized as the enzyme responsible for haem cleavage, with the release of CO, free iron (Fe^2+^) and biliverdin [4, 5].

There are two genetically distinct isozymes for HO: an inducible form haem-oxygenase-1 (HO-1) and a constitutively expressed form haem oxygenase-2 (HO-2). HO-1 occurs mainly in spleen, liver or bone marrow, and tissues that degrade senescent red blood cells; under conditions of haemolysis its activity dramatically increases. Higher levels of HO-2 occur mainly in testes and central nervous system [6]. Increase expression of HO-1 is associated with biological responses to several sources of stress, namely, oxidative stress, hypoxia, hyperoxia, misfolded protein response, hyperthermia, tumour promoter, ultraviolet radiation, and so forth. Concomitant with the increasing importance of HO activity in biological systems, CO is largely recognized as a homeostatic and cytoprotective molecule [7, 8]. Stimulation of endogenously generated CO and/or low doses of applied CO have shown to exert remarkable beneficial biological effects in many tissues: anti-inflammatory, antiapoptotic, antiproliferative and antiatherogenic. Three main areas of potential therapeutic applications have been extensively studied: cardiovascular diseases, inflammatory disorders, and organ transplantation [7], including the creation of several patents [9]. In the moment there are two clinical trials phase II on CO gas inhalation-based therapy: for treating patients with intestinal paralysis after colon surgery, for prevention of postoperative ileus (NCT01050712), and for the improvement of tolerability in patients receiving kidney transplants (NCT00531856).

**Figure 1:**
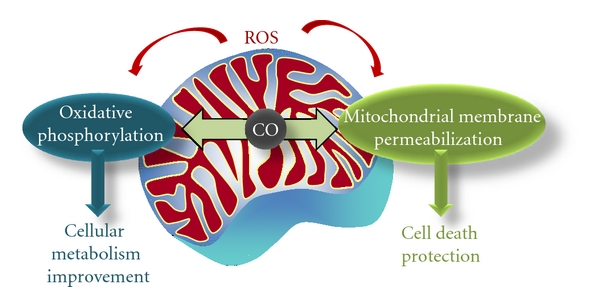
Two main aspects are involved in CO's cytoprotective role targeting mitochondria: modulation of cell metabolism by improvement of oxidative phosphorylation and inhibition of cell death by preventing mitochondrial membrane permeabilization.

The use of CO for therapeutic purposes presents two main advantages: (i) it is an endogenous product and the organism is fully adapted to and (ii) CO is not metabolized and reversibly binds to its molecular targets, which makes the pharmacokinetic much simpler. Due to its therapeutic potential, large efforts have been initiated in the last years to develop new ways to deliver this gas to specific tissues and organs. The main example is CO-releasing molecules (CORMs). CORMs are organic and organometallic compounds, able to deliver CO in a timely and tissue-specific manner, permitting a significant reduction in carboxyhaemglobin formation and toxicity [7, 9, 10]. In this paper, the carbon monoxide influence on cellular and tissue homeostasis by its direct action on mitochondria is emphasised, in particular on two aspects: cell metabolism and cell death control (Figure 1).

## 2. Chemistry of Carbon Monoxide

In biological systems, CO binds almost exclusively to transition metals, namely, iron, manganese, vanadium, cobalt, tungsten, copper, nickel, and molybdenum, which are present in structural and functional proteins [10]. The metal centre can interact with ligands (usually gases: CO, NO, and O_2_) modifying protein activity. The number of molecules targeted by CO in mammals is very limited; the majority are haem-containing proteins, whose function is regulated by the iron of this prosthetic group. Iron is involved in the regulation of protein function by being part of haem structure. In contrast to NO, that can bind to Fe^3+^ and Fe^2+^, CO is only able to accept electrons from Fe^2+^, which promotes a selectivity of CO-targeted haem proteins [10, 11]. Carbon monoxide presents high affinity for binding to haemoglobin and myoglobin, which competes with oxygen and compromises its delivery into tissues, causing hypoxia. Another member of globin superfamily is neuroglobin (Ngb), which is predominantly expressed in neurons and confers neuroprotection against hypoxic-ischemic injury [12]. Although the exact Ngb role is yet to be disclosed, its possible function includes O_2_ storage and transport or detoxification of ROS and NO. Furthermore, CO binding to the haem centre of Ngb leads to conformational changes and cellular signalling [13].

CO also activates soluble guanylate cyclase (sGC) and nitric oxide synthase (NOS), but higher levels of CO are usually required and its physiological role is not yet clarified. Finally, the last mitochondrial electron transport chain complex, cytochrome c oxidase appears as another potential binding target for CO *in vivo* [14].

Despite the biological functions associated to CO *in vivo* and the existence of several proteins capable for binding CO *in vitro*; it is still a matter of discussion: the actual physiological target of CO.

## 3. Carbon Monoxide and Cytochrome* c *Oxidase

Mitochondrion regulates cell metabolism, being the main energy source, responsible for most of cellular ATP production *via* oxidative phosphorylation. Mitochondrial membrane permeabilization is also tightly involved in cell death modulation and participates in signal transduction cascades, since several signaling factors are released from mitochondrial inter-membrane space. In 1970, it was first claimed that CO's toxicity could be derived from its targeting and binding to the mitochondrial haem-protein cytochrome *c* oxidase (COX) [15]. Cytochrome *c* oxidase is the final electron acceptor of the mitochondrial electron transport chain, catalysing the oxidation of ferrocytochrome *c* by gaseous oxygen.

Binding of CO to COX (cytochrome a, a3) was followed in rat brain by reflectance spectrophotometry. In rat exposed to CO (up to around 70% of carboxyhaemglobin levels in the blood) cytochrome a, a3 absorption (605 nm) increased in the parietal cortex, but no data on COX enzymatic activity were revealed [16, 17]. 90 minutes after ending CO exposure, cytochrome oxidation state improved to 80% of control under normobaric oxygen exposure and recovered completely under hyperbaric oxygen exposure [17]. It is worthy of note that 70% of carboxyhaemglobin levels is very high and exceeds the limit of toxicity. Usually, the used CO concentrations for animal experiments (~250–500 ppm) reach around 10 to 20% of carboxyhaemglobin levels.

In isolated mitochondria from human muscle, CO partially prevented COX activity at 50, 100, and 500 ppm, while no effect was obtained in respiratory complexes I, II, or III [18]. In mouse leukaemic monocyte macrophage cell line, 1 h of CO exposition (250 ppm) decreased COX activity, which was assessed in permeabilized cells [19]. In contrast, cytochrome *c* oxidase presented a two-step response to low concentrations of CO saturated solutions (10 *μ*M) in isolated mitochondria from mouse liver [20] and from rat brain [21]. In the first minutes (5 to 10) COX activity was partially prevented, while after 30 minutes its activity increased. Furthermore, COX-specific activity also increased whenever intact astrocytes were treated with 50 *μ*M of CO after 3 or 24 h [21].

These apparent controversial data can be due to different tested CO concentrations, period of gas exposure, and the time point for assessing cytochrome c oxidase activity. Still, CO effect on COX activity is also dependent on the system oxygen concentration. Indeed, CO competes with oxygen for binding to cytochrome *c* oxidase. However, the relative CO-O_2_ affinity (M*) is about 220 for haemoglobin and 20–25 for myoglobin while it is close to unit for cytochrome *c* oxidase [22]. Thus, the acute toxicity triggered by exogenous CO is mostly due to its high affinity for haemoglobin, limiting tissue oxygenation. Accordingly, CO capacity to bind to COX is highly dependent on oxygen concentration [14, 23]. Thus, another factor that might influence the distinct COX activity responses to CO found in the literature is the presence of oxygen concentration.

Accordingly, Fukuda et al. have shown that activation of hypoxia-inducing factor-1 (HIF-1) was involved in the regulation of cytochrome *c* oxidase subunits for optimizing the efficiency of mitochondrial respiration [24] and in macrophages CO-activated HIF-1 without increasing the rate of glycolysis [25]. Thus, one can speculate that CO improves COX activity by activation of HIF-1.

Finally, unlike NO and H_2_S that are readily metabolized by oxidative processes within COX, CO oxidation is too slow to be physiologically relevant as a substrate for COX [14].

## 4. Carbon Monoxide and Mitochondrial Respiration

An indirect way for assessing CO effect on mitochondria and on COX activity is by following cellular oxygen consumption. Under normoxia (21% of oxygen in gaseous phase), exogenous CO application or endogenous CO (generated by overexpression of HO-1 or by lipopolysaccharide induced HO-1 expression) slightly inhibited cell respiration, but it is not clear the physiological importance of this inhibition. In contrast, endogenous or exogenous CO considerably decreased cellular respiration under hypoxia conditions (1% of oxygen) [23]. Thus, tissue hypoxia and CO appeared to have a synergistic effect on COX inhibition. It can be speculated that CO significantly inhibits COX only when tissue oxygen delivery is already compromised by the presence of high levels of carboxy-haemoglobin and carboxymyoglobin. Furthermore, under low O_2_ concentration the electron transport chain is in a more reduced state, which is a more favourable state for CO to bind, since CO has the ability for binding to reduced iron [10, 26].

In isolated mitochondria from kidney, respiratory control index was assessed immediately after adding three different CO-releasing molecules (CORM-2, CORM-3, and CORM-A1) at 10, 50, and 100 *μ*M. Oxygen consumption was measured in the presence (state 3) or absence (state 4) of ADP; the ratio between state 3 and 4 is the respiratory control index (RCR) and indicates the tightness of the coupling between respiration and phosphorylation. At this early time point CO decreased respiratory control index [27]. In contrast, during reperfusion, after kidney cold storage, CO is released by CORM-3 or CORM-A1 increased renal mitochondrial respiration, improving its function [28].

Iacono et al. have claimed that CO limits excessive mitochondrial ROS production and avoids oxidative stress by inducing a mild-uncoupling state [29]. This hypothesis was based on the following data. First, low concentrations (up to 20 *μ*M) of CORM-3 weaken the coupling between ATP production and respiration. In the absence of exogenous ADP (state 4), CO-treated heart-isolated mitochondria presented an increase on oxygen consumption. In this situation, complex II appears to be the target of CO since inhibition of complex II (malonate addition) reverted the CO-induced augmentation of oxygen consumption. Second, in the presence of ADP (state 3), 100 *μ*M of CORM-3 decreased oxygen consumption, which was claimed to be due to complex IV inhibition. Third, CORM-3 at 20 or 100 *μ*M decreased mitochondrial membrane potential (ΔΨm). This decrease was prevented by the addition of inhibitors for uncoupling respiration proteins (UCP) and for adenine nucleotide transporter (ANT), indicating that CO might open UCP and/or ANT for providing a mild uncoupling state. Taken all together, CO-induced cytoprotection was correlated with a mitochondrial mild uncoupling stimulation, which decreases excessive and toxic mitochondrial ROS production [29]. However, all data were obtained using isolated heart mitochondria and the actual physiological role remains to be disclosed. Still, only an early-response of CO was assessed, thus further studies are necessary to clarify the CO late response on mitochondrial mild uncoupling.

In a distinct experimental approach, astrocytes isolated from cortex have received one brief exposition to CO (by addition of CO-saturated solution at 50 *μ*M final concentration from which the gas diffuses rapidly) and cell-specific oxygen consumption was assessed during 36 h. Indeed, brief exposition to CO increased cellular oxygen consumption in intact astrocytes, which was justified by an improvement of mitochondrial respiratory chain and oxidative metabolism [21].

Accordingly to the two-step time response of cytochrome *c* oxidase activity to CO; whereas it was observed an early or late response corresponding to a decrease or an increase on COX activity, respectively [20, 21]. Lancel et al. have also found that mitochondrial respiration has two distinct responses to CO depending on its concentration [30]. In heart-isolated mitochondria, 0.5 and 1 *μ*M of CORM-3 increased respiratory control ratio (RCR) and mitochondrial transmembrane potential (ΔΨm), while at 5 and 10 *μ*M there is a decrease in RCR and ΔΨm [30]. Once again, experimental conditions (concentration or period of exposure) might change the tight mitochondrial balance and modulation of oxidative metabolism, namely, the influence of CO on COX activity, mitochondrial respiration, or cellular oxygen consumption.

## 5. Carbon Monoxide and Mitochondrial Biogenesis

CO effect on mitochondria is not limited to organelle functioning but also involves modulation of their population. In fact, mitochondrial biogenesis stimulation appears to be one of the CO biological functions in several models. In the heart and in isolated cardiomyocytes, CO triggered mitochondrial biogenesis, which was activated by the expression of nuclear respiratory factor 1 (NRF-1), binding to mitochondrial transcription factor 3 (TFAM-3) [31]. CO activation of mitochondrial biogenesis involved both guanylate cyclase and prosurvival kinase Akt/PKB and occurred in a hydrogen-peroxide-(H_2_O_2_-) dependent manner. Cell transfection with mitochondrial-targeted catalase, which scavenges mitochondrial H_2_O_2_, prevented CO-induced mitochondrial DNA replication [31]. Likewise, heam oxygenase modulates cardiac mitochondrial biogenesis via gene expression and nuclear translocation of nuclear factor erythroid-2-related factor 2 (Nrf2), which upregulates expression of nuclear respiratory factor 1 (NRF-1) [32]. Furthermore, the same authors have shown that CO/haem oxygenase system prevented murine doxorubicin cardiomyopathy by reversing mitochondrial biogenesis inhibition [33]. In another pathological model, peritonitis-induced sepsis, carbon monoxide can rescue mice from death by providing energetic metabolic support *via* activating mitochondrial biogenesis [30]. CORM-3 treatment activated mitochondrial biogenesis and induced an increase on RCR, ΔΨm, mitochondrial H_2_O_2_ concentration, and mitochondrial DNA level. However, none great effect on pro- or anti-inflammatory markers (TNF-*α* or IL-10) was observed, only after 48 h a slight increase on IL-10 was found [30]. More recently, in another rodent model of sepsis, induction of HO-1 coupled activation of mitochondrial biogenesis to anti-inflammatory cytokine expression, such as IL-10 or sIL1-Ra [34]. A boost of CO exposure, using saturated solutions, also activated mitochondrial biogenesis in astrocytes from rat cortex [21]. Finally, in human skeletal muscle exposition to low concentrations of CO (1 h/day at 100 ppm during 5 days) stimulated mitochondrial biogenesis involving regulation of the mitochondrial DNA transcriptome [35].

Therefore, activation of mitochondrial biogenesis is another biological function conferred by carbon monoxide for the improvement of cell metabolism and for providing cytoprotection.

## 6. Carbon Monoxide, Metabolism, and Energy Status

Based on the facts that CO increases mitochondrial population, modulates mitochondrial respiration, and can regulate mitochondrial respiratory complexes, it is not surprising the great influence of CO on cellular metabolism and energetic status.

Carbon monoxide improved cardiac energy in a model of ischemia and reperfusion in pigs, preventing edema and apoptosis [36]. Pigs were ventilated for 2 h with 250 ppm CO and then subjected to cardiopulmonary bypass. CO treatment had no effect on ATP/ADP ratio before ischemia, but after reperfusion CO resulted in higher levels of ATP. Likewise, phosphocreatine, a high-energy phosphate reserve in the cell, which facilitates intracellular high-energy phosphate transport, slightly decreased with CO treatment before ischemia. Yet, after reperfusion, the phosphocreatine levels in CO-treated hearts were significantly higher than in nontreated animals. Thus, CO improved energetic status and prevented cardiac tissue damage by edema and cell death [36]. Likewise, in a similar model of heart ischemia in pigs, CO was administrated at concentrations with the goal to obtain up to 5% of carboxyhaemglobin in the blood. In CO-treated animals the lactate production/glucose consumption ratio decreased, meaning that higher amounts of pyruvate entered and were metabolized by TCA cycle and a decrease on glycolytic metabolism was observed [37]. In human hepatocytes or primary culture of mouse hepatocytes, exogenous or heam-oxygenase-1-derived CO stimulated ATP generation [38, 39]. In this model, activation of soluble guanylyl cyclase (sGC) was the proposed pathway for ATP generation increase, and the strength of p38 MAPK activation was correlated with the availability of ATP. Furthermore, 1 h of CO inhalation (at low concentrations: 100 to 500 ppm) enhanced sGC activity and ATP generation in mouse liver, improving the survival in mice death after initiation of fulminant hepatitis [38]. Finally, in primary culture of astrocytes, CO treatment also stimulated ATP generation by improving oxidative metabolism [21].

## 7. Carbon Monoxide and Cytoprotection

In 2000 Brouard et al. demonstrated for the first time the antiapoptotic property of the system HO-1/CO in endothelial cells, whose mechanism of action was dependent on the activation of the p38 mitogen-activated protein kinase (MAPK) signaling transduction pathway [40]. Since then and in many different models of lung, brain, smooth muscle cells, liver or endothelial cells low concentrations of exogenous CO, or haem oxygenase-derived CO have shown to confer resistance against cell death [6–8].

In hepatocytes, CO prevented TNF-*α*-induced apoptosis via inhibiting caspase-8 [41] or CO prevented oxidative stress-induced apoptosis by inhibition of p54 JNK isoform [42]. Likewise, CO-induced hepatocyte NF-kappaB activation and apoptosis protection presenting reactive oxygen species (ROSs) generation as signaling molecules [43]. Low levels of exogenous CO attenuated anoxia-reoxygenation-induced lung endothelial cell apoptosis via activation of p38 MAPK and STAT 3, which prevented caspase-3 activation [44, 45]. Additionally, CO protected against hyperoxia-induced endothelial cell apoptosis by preventing excessive ROS formation, bid activation, mitochondrial translocation of Bax, cytochrome *c* release, and caspase-9/3 activation [46]. In neuronal primary cultures, CO prevented glutamate-induced apoptosis through ROS generation and activation of sGC and inducible nitric oxide synthase (iNOS) [47]. Carbon monoxide blocks apoptosis in vascular smooth muscle cells, in part, by activating the cGMP signaling pathway [48, 49].

Thus, cytoprotection, namely, prevention of apoptosis, jointly with anti-inflammatory properties, are the two most explored biological functions of CO.

## 8. Role of Mitochondria and ROS Signalling on Costimulated Cytoprotection

Despite the existence of a long list of publications demonstrating cytoprotection of CO, the role of mitochondria on CO-induced cell death modulation is still poorly explored. First evidence showing mitochondria involvement on CO mode of action raised from the crucial role of ROS as signalling molecules, which were generated at mitochondrial level. Experimental approaches using respiratory deficient *ρ* ^0^ cells revealed the importance of mitochondrial generated ROS for CO to prevent inflammation in macrophages [19, 25] and to inhibit cell death in hepatocytes [43]. Furthermore, antioxidant addition has also indicated the importance of ROS signalling in several models, by reversing biological functions of CO, such as: (i) cardioprotection *via* inhibition of L-type Ca^2+^ channels [50], (ii) inhibition of apoptosis in astrocytes and neurons [47, 51], (iii) antiproliferative effect in airway smooth muscle cells [52], and (iv) induction of mitochondrial biogenesis in cardiomyocytes [31]. Thus, low levels of ROS appear as important signalling molecules in CO biology. It is worthy of note that although presenting ROS as essential signalling molecules, CO also limits mitochondrial oxidative stress and excessive ROS generation, for instance by inducing a mild uncoupling effect [29]. How CO controls the tight balance between generation of low signalling and high toxic levels of ROS is still a matter of debate.

**Figure 2:**
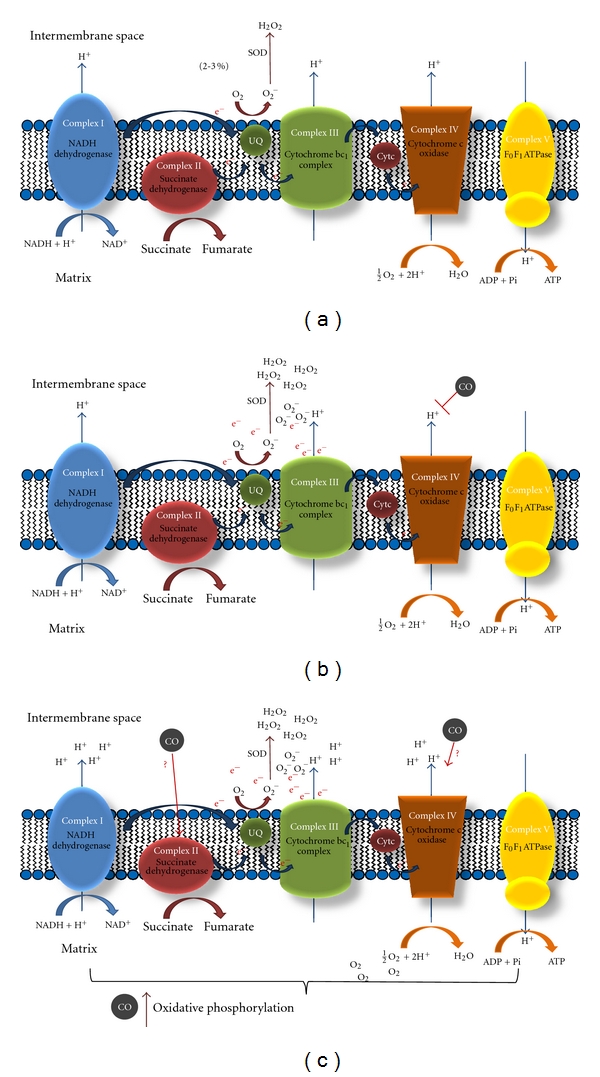
Proposed models for CO action on mitochondria. (a) Under physiological conditions, 1–3% of the consumed oxygen is incompletely reduced to anion superoxide (O_2_^−^), which is rapidly converted into hydrogen peroxide (H_2_O_2_) by the superoxide dismutase present in the mitochondrial matrix. (b) The most accepted hypothesis for CO to generate mitochondrial ROS is based on partially and/or reversely inhibition of cytochrome *c* oxidase (complex IV), leading to electron accumulation at complex III level, which facilitates anion superoxide generation. (c) Since low doses of CO also improve mitochondrial respiration, it can be speculated that CO induces mitochondrial ROS generation because oxidative phosphorylation is accelerated. The exact mitochondrial target is not fully understood, but complexes II and IV are strong candidates.

Several hypotheses exist in the literature for the mode CO generates signalling ROS. Under physiological conditions, mitochondria continuously produce low levels of anion superoxide (O_2_^−^) as a byproduct of oxidative phosphorylation since 1–3% of the consumed oxygen is incompletely reduced to O_2_^−^. Anion superoxide is rapidly converted into hydrogen peroxide (H_2_O_2_) by the superoxide dismutase present in the mitochondrial matrix (Figure 2(a)) [53]. Interestingly, hydrogen peroxide is much more stable than anion superoxide, is capable of diffusing through biological membranes [54] and is a potent signalling molecule [55]. A growing body of evidence suggests that ROSs are physiologically generated at the level of complexes I and III of the mitochondrial respiratory chain. The most accepted hypothesis for CO to generate mitochondrial ROS is supported by CO capacity of (partially and/or reversely) inhibiting cytochrome c oxidase (complex IV), leading to electron accumulation at complex III level, which facilitates anion superoxide generation (Figure 2(b)) [56]. Still, one can also speculate that CO induces mitochondrial ROS generation by accelerating mitochondrial respiration and oxidative phosphorylation, increasing the amount of oxygen that is not totally reduced into water (Figure 2(c)). The exact mitochondrial target and mode of action for CO to accelerate mitochondrial respiration and functioning is still a matter of debate: complex IV is the strongest candidate [56], but complex II also seems to be involved [29]. Furthermore, CO also stimulates mitochondrial biogenesis, which can cause augmentation of ROS by increased mitochondrial population.

## 9. Mitochondrial Membrane Permeabilization and Carbon Monoxide

Apoptosis occurs via two distinct pathways: an extrinsic pathway (relying on cell surface membrane receptors) and an intrinsic pathway, which is triggered by several conditions of intracellular stress, leading to mitochondrial membrane permeabilization (MMP). In many models, MMP induces (i) mitochondrial transmembrane potential dissipation, (ii) respiratory chain uncoupling, (iii) ROS overproduction, (iv) ATP synthesis arrest, and (v) the release of several death-regulating molecules (activating proteases and nucleases), making the cell death process irreversible [57]. Despite the vast amount of publications concerning cell death prevention by CO, very few data are available for CO's direct implication on MMP control.

In 2006 Piantadosi et al. performed an interesting study on mitochondrial permeability pore transition, oxidative stress, and carbon monoxide. Rats were continuously exposed to 50 ppm of CO for 1, 3, or 7 days. At day 1 and 3, CO has increased a prooxidative state at liver mitochondria, which were more sensitive to Ca^2+^ for opening a pore on mitochondrial membrane. While at day 7, continuously exposure to CO prevented MMP in liver mitochondria by increasing expression of antioxidant enzymes: haem oxygenase-1 (HO-1) and manganese superoxide dismutase (SOD-2) [58]. Thus, CO-induced preconditioning generates an anti-oxidant state, which promotes liver resistance against MMP.

The direct role of CO on targeting mitochondria was evaluated in isolated nonsynaptic mitochondria from rat cortex. MMP was induced by Ca^2+^ and carboxyatractyloside treatment followed by the assessment of: (i) loss of mitochondrial potential, (ii) the opening of a ~800 Da pore through the inner membrane, (iii) swelling, and (iv) cytochrome *c* release [51]. CO inhibition of these four events was reversed by the addition of an antioxidant, *β*-carotene, indicating that ROS are important signalling molecules at mitochondrial level. Moreover, CO induced slight increase in mitochondrial-oxidized glutathione, which triggered ANT glutathionylation and enhanced its ATP/ADP translocation activity through the inner membrane. Thus, CO directly prevented MMP and its consequent astrocytic cell death and accelerated ATP/ADP transport through mitochondria. Accordingly, upregulation of HO-1 (by overexpression or cobalt protoporphyrin addition) is associated with an increase on mitochondrial transport carriers (carnitine, deoxynucleotide, and ATP/ADP carriers) and cytochrome *c* oxidase activities in experimental diabetes [59]. In isolated liver mitochondria from mouse, pretreatment with CO also inhibited mitochondrial swelling, depolarization, and the opening of a pore through the inner membrane, in a ROS-dependent manner [20]. In addition, cytochrome c oxidase transiently responded to low concentrations of CO by decreasing its activity in the first 5 minutes after treatment, while later on there was an increase of COX activity detected up to 30 minutes [20].

It is worth of note that CO stimulates the antiapoptotic protein Bcl-2 expression in lung and cerebral ischemia models [60, 61], and Bcl-2 can translocate into mitochondrial membranes for preventing their permeabilization and cell death. Thus, CO is also capable for preventing MMP by indirectly acting on Bcl-2 expression levels and subcellular localization.

## 10. Final Remarks

 “The dose makes the poison”, carbon monoxide, a simple and small molecule, known to be toxic, presents beneficial pleiotropic effects. Low concentrations of CO are able to activate distinct endogenous cell defence mechanisms: antiapoptosis, anti-inflammation, antiproliferation, metabolism improvement, cardioprotection, and so forth. By preconditioning the cells CO is a cytoprotective factor, and mitochondria appear as the main cellular targets. In addition, CO is an endogenous gaseoustransmitter, which is physiologically generated in response to several types of stress.

Diverse, and apparently controversial, data are available in the literature concerning CO model of action on mitochondria, in particular on cytochrome c oxidase activity and on oxygen consumption. One can speculate that CO biological activity might depend on two main factors: period of CO exposure and gas concentration, giving rise to distinct responses. Furthermore, different CO sources increase the system complexity and do not facilitate data comparison. For instance, CO can be applied by different modes: (i) one single burst of CO (CO-saturated solutions fast gas diffusion), (ii) gas exposition (continuously application during the period of exposition), and (iii) CO-releasing molecules. Depending on the used CORM and its specific molecular characteristics, these molecules can be slow or fast CO releasers or can differently respond to tissues or to a physiological situation, such as an increase on oxidative stress.

**Figure 3:**
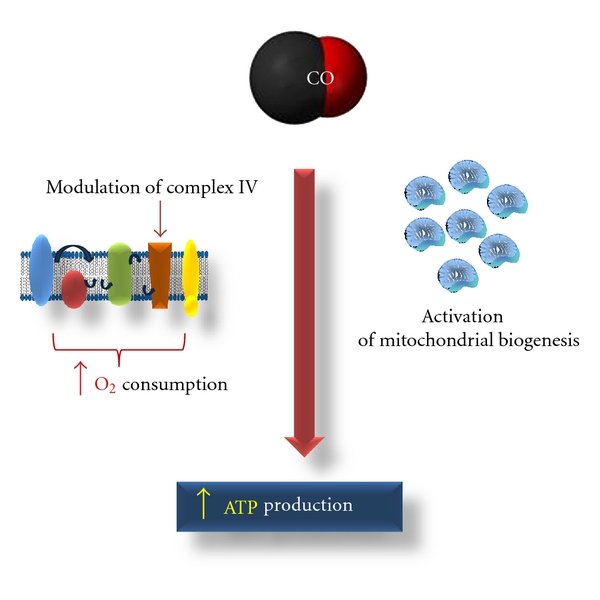
CO controls mitochondrial functioning and improves cellular energetic state (increased ATP generation) by two main ways: enhance of oxidative phosphorylation and induction of mitochondrial biogenesis.

In conclusion, CO controls mitochondrial functioning and oxidative metabolism, improving cellular energetic state, by modulation: COX activity, oxygen consumption, mitochondrial biogenesis, and ROS generation (Figure 3). Additionally, CO also prevents cell death: (i) by directly targeting mitochondria and inhibiting mitochondrial membrane permeabilization (MMP), (ii) by increasing antiapoptotic gene expression, such as Bcl-2, which also prevents MMP, or (iii) by interacting with the apoptosis-inducing cytochrome c-cardiolipin complex and inhibiting caspase activation [62].

The future in CO research field lays on the disclosure of the cross-talk between cell death and cell metabolism modulation (Figure 1). Mitochondria are the key organelle involved in the control of both cellular events. Thus, searching for the physiological mitochondrial target of CO and the biochemical and cellular mechanisms involved is crucial for the development of this gaseoustransmitter as a novel therapeutic agent.

## Abbreviations

ANT: Adenosine nucleotide translocase
ATP: Adenosine triphosphate
O_2_^−^: Anion superoxide
CO: Carbon monoxide
CORM: Carbon monoxide releasing molecules
COX: Cytochrome *c* oxidase or complex IV
HIF-1: Hypoxia-inducing factor-1
HO: Haem oxygenase
H_2_O_2_: Hydrogen peroxide
MMP: Mitochondrial membrane permeabilization
NRF-1: Nuclear respiratory factor 1
Nrf-2: Nuclear factor erythroid-2 related factor 2
ppm: Parts per million
RCR: Respiratory control index
ROS: Reactive oxygen species
sGC: Guanylate cyclase
TFAM: Mitochondrial transcription factor 3
UCP: Uncoupling respiration proteins
ΔΨm: Mitochondrial membrane potential.
